# Clinical features of, and risk factors for, severe or fatal COVID-19 among people living with HIV admitted to hospital: analysis of data from the WHO Global Clinical Platform of COVID-19

**DOI:** 10.1016/S2352-3018(22)00097-2

**Published:** 2022-05-10

**Authors:** Silvia Bertagnolio, Soe Soe Thwin, Ronaldo Silva, Sairaman Nagarajan, Waasila Jassat, Robert Fowler, Rashan Haniffa, Ludovic Reveiz, Nathan Ford, Meg Doherty, Janet Diaz

**Affiliations:** a Department of Global HIV, STI & Hepatitis Programmes, WHO, Geneva, Switzerland; b Department of Sexual and Reproductive Health and Research, WHO, Geneva, Switzerland; c Department of Country Readiness Strengthening, Health Emergencies Programme, WHO, Geneva, Switzerland; d Departments of Internal Medicine and Pediatrics, SUNY Downstate Medical Center, New York, USA; e National Institute for Communicable Diseases, Johannesburg, South Africa; f Right to Care, Pretoria, South Africa; g Department of Medicine, Sunnybrook Health Sciences Centre, Toronto, Canada; h Mahidol Oxford Tropical Medicine Research, Nuffield Department of Medicine, University of Oxford, Oxford, UK; i National Intensive Care Surveillance-MORU, Colombo, Sri Lanka; j Department of Evidence and Intelligence for Action in Health, Incident Management Systems, Pan American Health Organization, Washington DC, USA

## Abstract

**Background:**

WHO has established a Global Clinical Platform for the clinical characterisation of COVID-19 among hospitalised individuals. We assessed whether people living with HIV hospitalised with COVID-19 had increased odds of severe presentation and of in-hospital mortality compared with individuals who were HIV-negative and associated risk factors.

**Methods:**

Between Jan 1, 2020, and July 1, 2021, anonymised individual-level data from 338 566 patients in 38 countries were reported to WHO. Using the Platform pooled dataset, we performed descriptive statistics and regression analyses to compare outcomes in the two populations and identify risk factors.

**Findings:**

Of 197 479 patients reporting HIV status, 16 955 (8·6%) were people living with HIV. 16 283 (96.0%) of the 16 955 people living with HIV were from Africa; 10 603 (62·9%) were female and 6271 (37·1%) were male; the mean age was 45·5 years (SD 13·7); 6339 (38·3%) were admitted to hospital with severe illness; and 3913 (24·3%) died in hospital. Of the 10 166 people living with HIV with known antiretroviral therapy (ART) status, 9302 (91·5%) were on ART. Compared with individuals without HIV, people living with HIV had 15% increased odds of severe presentation with COVID-19 (aOR 1·15, 95% CI 1·10–1·20) and were 38% more likely to die in hospital (aHR 1·38, 1·34–1·41). Among people living with HIV, male sex, age 45–75 years, and having chronic cardiac disease or hypertension increased the odds of severe COVID-19; male sex, age older than 18 years, having diabetes, hypertension, malignancy, tuberculosis, or chronic kidney disease increased the risk of in-hospital mortality. The use of ART or viral load suppression were associated with a reduced risk of poor outcomes; however, HIV infection remained a risk factor for severity and mortality regardless of ART and viral load suppression status.

**Interpretation:**

In this sample of hospitalised people contributing data to the WHO Global Clinical Platform for COVID-19, HIV was an independent risk factor for both severe COVID-19 at admission and in-hospital mortality. These findings have informed WHO immunisation policy that prioritises vaccination for people living with HIV. As the results mostly reflect the data contribution from Africa, this analysis will be updated as more data from other regions become available.

**Funding:**

None.

**Translation:**

For the French translation of the abstract see Supplementary Materials section.

## Introduction

As of April 28, 2022, more than 509 million COVID-19 cases and 6·2 million deaths were reported globally.^1^ Low-income and middle-income countries (LMICs), notably Brazil, India, and South Africa, have reported high numbers of COVID-19 cases. At the same time, 37·7 million people are living with HIV worldwide and 1·5 million became newly infected with HIV in 2020, the majority (67%) of whom are in sub-Saharan Africa.^2^ Characterising populations at increased risk of severe or fatal COVID-19 is critical for appropriate prioritisation of interventions, particularly in areas where resources are limited, the health-care system is stretched, there is a high prevalence of communicable diseases such as HIV and tuberculosis affecting the burden and severity of COVID-19, and where vaccine coverage is unacceptably low. As of December, 2021, fewer than 7% of people in Africa had received at least one dose of a COVID-19 vaccine, but this proportion is progressively increasing (23% as of 28 April 2022).^1^

People living with HIV have underlying immune dysregulation,^3^ putting them at risk for opportunistic infections,^4^ autoimmune diseases,^5^, ^6^ and cancer.^5^ Overall, people living with HIV are more prone to disordered T-lymphocyte and B-lymphocyte, and cytokine–interferon responses, and polyclonal (yet ineffective) antibody production,^7^ and are more commonly affected by non-communicable diseases, including diabetes and cardiovascular diseases.^8^ All these factors might—in principle—put them at higher risk for severe or fatal disease when co-infected with SARS-CoV-2. However, before this study, the evidence to support this hypothesis was sparse and inconsistent, with most analyses based on small and geographically limited samples.^9^, ^10^, ^11^, ^12^, ^13^, ^14^, ^15^ A study that analysed data from sites across the UK found that people living with HIV had an increased risk of mortality,^15^ whereas studies from Belgium^16^ and Spain did not.^17^, ^18^ Data from within regions have also been conflicting. In two studies from New York state (USA), one found that people living with HIV are at increased risk for severe disease requiring hospitalisation,^19^ and the other showed no overall increased risk for intensive care unit (ICU) admission, intubation, or mortality.^20^ Early single-centre cohort studies and meta-analyses from high-income countries with small numbers of people living with HIV did not find HIV to be a risk factor for severe COVID-19. However, three meta-analyses have now reported an increased risk of mortality among people living with HIV.^21^, ^22^, ^23^ Additionally, recent studies found an increased risk of severe outcomes among people living with HIV, particularly in those with detectable viremia^24^ and increased odds of death in people living with HIV, with older age and male sex being risk factors.^25^ Recent data from South Africa also found that HIV infection was an independent risk factor for in-hospital mortality.^26^

**Research in context**

**Evidence before this study**

PubMed was searched for studies published between Feb 1, 2020, and July 31, 2021, in English, using the search strings “HIV (and) COVID-19, people living with HIV (and) COVID-19, HIV (and) COVID-19 (and) severity, and HIV (and) COVID-19 (and) mortality (or) death”. Evidence regarding the risk of adverse COVID-19 outcomes in people living with HIV has shown conflicting findings across observational studies and geographical regions, and estimating the extent to which this risk is modified by other factors has been limited by small sample sizes or geographic restraints. Studies from the UK and South Africa found that people living with HIV have an increased mortality risk for COVID-19, but similar conclusions were not drawn from studies from Belgium or Spain. Although early single-centre cohort studies and meta-analyses of data from high-income settings with small numbers of cases did not find HIV to be a risk factor for severe COVID-19, larger population cohorts and meta-analyses of larger datasets found that people living with HIV had a moderately increased risk of mortality. Broader geographical representation is required to expand understanding on how HIV infection impacts clinical outcomes secondary to hospitalisation with COVID-19.

**Added value of this study**

To our knowledge, this is the largest analysis to date exploring the association between HIV infection and clinical outcomes in people hospitalised with COVID-19 using individual patient-level data. The strength of the WHO Clinical Platform lies in the wide representation of contributing countries (38 countries, with 22 from low-income and middle-income countries), and in the collection of individual-level data of 16 955 people living with HIV and 180 524 people who were HIV-negative using standardised definitions and tools.

**Implications of all the available evidence**

Underlying conditions were more frequently observed in people living with HIV, stressing the need for this population to stay as healthy as possible, regularly take their antiretroviral therapy medications, and prevent and manage underlying conditions that can increase the risk of adverse outcomes. The increased risk of poor outcomes in people living with HIV hospitalised for COVID-19 should be considered when prioritising SARS-CoV-2 vaccination among vulnerable groups. These findings have informed the WHO Strategic Advisory Group of Experts on Immunization roadmap for prioritising the use of COVID-19 vaccines in the context of limited supply, which now includes HIV infection among the chronic conditions to consider in vaccine prioritisation. Countries should consider including people living with HIV as a priority group for COVID-19 vaccination according to their epidemiological context. Data contribution to the WHO Platform is ongoing, to further explore the reasons for adverse outcomes beyond the variables assessed in this dataset, including the potential influence of CD4 cell counts.

To inform public health interventions around the prevention and management of COVID-19, our study aimed to explain these conflicting results and generate more conclusive data on the association between HIV infection and severe or fatal COVID-19 among people admitted to hospitals globally, and particularly in sub-Saharan Africa.

In April, 2020, WHO launched the WHO Global Clinical Platform on COVID-19,^27^ which is a secure, web-based database of anonymised individual-level clinical data of hospitalised patients with suspected or confirmed COVID-19 from health facilities across the world. Using data from this platform, we assessed whether people living with HIV hospitalised with COVID-19 were at increased risk of severe or critical presentation on admission and of in-hospital mortality compared with hospitalised HIV-negative individuals with COVID-19, and investigated risk factors associated with severe or critical illness at hospital admission and in-hospital mortality among people living with HIV hospitalised for COVID-19.

## Methods

### Data sources

Ministries of Health, research networks, and health facilities were formally invited by WHO to contribute anonymised clinical data to the WHO Platform using a standardised Case Report Form (CRF)^27^ and data dictionary.

The WHO CRF contains a standardised set of variables to be collected on hospital admission, daily, and at the time of hospital discharge. Information includes demographics, pregnancy status, country, vital signs and other anthropometrics, underlying conditions, chronic medications, clinical features, laboratory testing, therapeutics, admission to an ICU, use of oxygen, use of mechanical ventilation, complications arising due to COVID-19, and clinical outcomes (discharge, death, transfer to another facility, or remaining hospitalised at the time of data entry).

### Study design and population

All patients admitted to a health-care facility with laboratory-confirmed or clinically suspected COVID-19 were eligible for inclusion. Cases were defined as severe or critical, according to a modified definition from WHO Clinical Management Guidelines of COVID-19,^28^ if they met one or more of the following conditions at hospital admission: SpO_2_ of less than 90%; respiratory rate of more than 30 breaths per min in adults and children older than 5 years (≥40 breaths per min in children aged 1–5 years, ≥50 breaths per min in children aged 2 years to 11 months, ≥60 breaths per min in children aged <2 months); received extracorporeal membrane oxygenation; admitted to ICU; received an inotrope or vasopressor; or received oxygen therapy or either invasive or non-invasive ventilation. Cases not meeting any of the conditions above were categorised as mild or moderate.

Data collection was retrospective, prospective, or both. Many facilities in LMICs were trained by WHO on data entry. Additionally, research networks, health facilities, and authors of published articles (identified through a rapid review of PubMed) were invited to share their datasets and data dictionaries. When definitions were consistent with the WHO CRF, variables were transferred to the WHO Clinical Platform.

The analysis plan was submitted to the WHO Ethical Review Committee, which granted a waiver from ethical review clearance because this was anonymised clinical surveillance. Ethical clearance was obtained, where necessary, by relevant institutional or national bodies.

### Statistical analysis

Descriptive and regression analyses were done to summarise demographic and clinical characteristics by HIV status and to evaluate their association with disease severity at hospital admission, and in-hospital mortality (primary outcomes). Records with missing data were excluded when determining distributions across outcome levels; χ^2^ tests and student t-tests were used to assess the relationship between clinical characteristics and primary outcomes. Multivariable logistic regression models using generalised estimating equations were fitted to evaluate whether HIV infection was an independent risk factor for severe or critical illness and proportional hazards models were fitted to evaluate whether HIV infection was a risk factor for in-hospital mortality. The models were adjusted in variance estimation for potential correlation for clustering at the country level.

Age, sex, and HIV status were included in the models a priori. Other covariates were considered for inclusion in the model when they were: reported in more than 80% of the cases; not highly correlated with other variables using a correlation matrix threshold of more than 0·8; independently associated with both the outcomes and HIV status at a p value of less than 0·10. Included covariates were retained in the final model if they were found to be significant at p values of less than 0·05 (appendix 2 pp 7–10).

Based on the above criteria, the following conditions were considered as potential covariates: asplenia, asthma, chronic cardiac disease, chronic kidney disease, chronic liver disease, chronic neurological disorder, chronic pulmonary disease, current smoking, diabetes, hypertension, malignant neoplasms, obesity, and tuberculosis. Tuberculosis was defined as current (active) or previous infection. We also considered a composite covariate based on number or burden of conditions (none, 1–2, ≥3 conditions) in a separate model.

In addition, using the same approach described above, we conducted a regression analysis to assess risk factors for disease severity at admission and in-hospital mortality among people living with HIV.

Lastly, we conducted exploratory subgroup analyses to assess the impact of geographical location, viral load, and antiretroviral therapy (ART) on mortality and severity.

All analyses were conducted in SAS version 9.4, or R version 3.6.3.

### Role of the funding source

There was no funding source for this study.

## Results

This analysis reflects the findings from 197 479 cases (16 955 people living with HIV and 180 524 people who were HIV-negative) submitted to the WHO Global COVID-19 Clinical Platform between Jan 1, 2020, and July 1, 2021, from 38 countries (figure; appendix 2 p 2 shows countries submitting >30 patients). 184 805 (93·6%) of the 197 479 admitted cases and 16 398 (96·7%) of 16 955 people living with HIV were hospitalised with laboratory-confirmed COVID-19 (179 886 [98·3%] of 182 911 and 16 149 [99·2%] of 16 283 people living with HIV in the African region). 6162 (97·2%) of 6330 people living with HIV with severe presentation at hospital admission and 3913 (98·4%) of 3849 people living with HIV who died had laboratory-diagnosed COVID-19.

**Figure:**
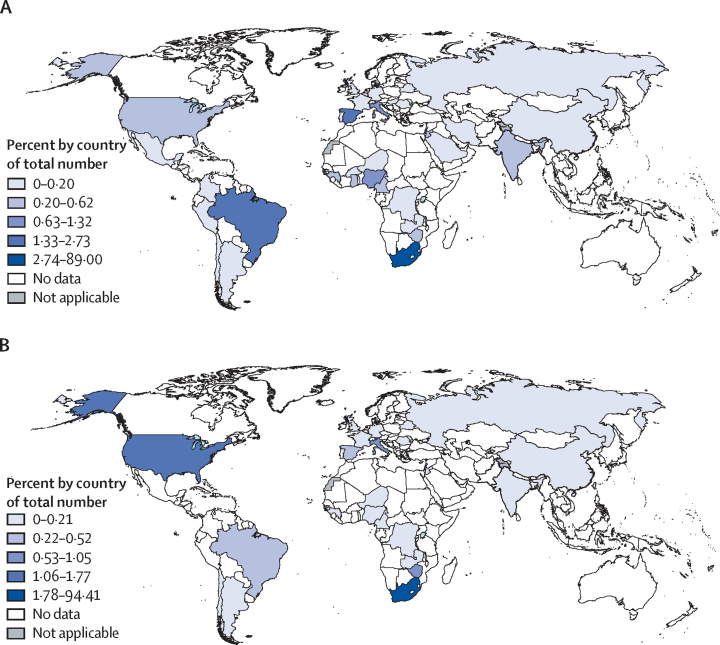
Countries contributing clinical data on COVID-19 hospitalised cases to the WHO Global Clinical Platform for COVID-19 (as of July 1, 2021) (A) 38 countries contributing clinical data on people living with HIV hospitalised with COVID-19: Argentina, Belarus, Belgium, Brazil, Burkina Faso, Cameroon, Chile, China, Colombia, Democratic Republic of Congo, Dominican Republic, France, Germany, Ghana, Guinea, Hungary, India, Iran, Italy, Jordan, Mexico, Niger, Nigeria, Panama, Peru, Portugal, South Korea, Romania, Russia, Saudi Arabia, Singapore, South Africa, Spain, Switzerland, USA, UK, Zambia, Zimbabwe. (B) 25 countries contributing clinical data on people living with HIV: Argentina, Belarus, Brazil, Cameroon, Chile, China, Democratic Republic of the Congo, Dominican Republic, France, Germany, Guinea, India, Italy, Jordan, Niger, Nigeria, Panama, Romania, Russia, South Africa, Spain, UK, USA, Zambia, Zimbabwe.

16 283 (96·0%) of 16 955 people living with HIV were from Africa, largely from South Africa (n=16 008). Other regions contributing data of people living with HIV were the Americas (n=395), European region (n=256), Western Pacific/South East Asia (n=20), and the Eastern Mediterranean Region (n=1). 10 166 (59·9%) of 16 955 people living with HIV reported ART information and 9302 (91·5%) received treatment.

Compared with people who were HIV-negative, people living with HIV were more likely to be female and younger than 45 years; the mean age was 45·5 years (SD 13·7), and 1253 (7·6%) were older than 65 years versus 47 237 (26·7%) of the people who were HIV-negative (p<0·0001; table 1). The presence of at least one underlying condition was more frequent among people living with HIV (60·8%) compared with people who were HIV-negative (46·0%). All conditions except hypertension, neurological disorders, obesity, and diabetes were more frequent in people living with HIV (table 1).

**Table 1:** Characteristics, underlying conditions, therapeutics, and outcomes of people hospitalised with suspected or confirmed COVID-19, by HIV status

	**Total (n=197** **197)**	**People living with HIV (n=16** **955)**	**HIV-negative (n=180** **524)**	**p value**
**Age group, years**				
≤18	6727	259 (1·5%)	6468 (3·7%)	<0·0001
>18–45	56 421	8177 (49·1%)	48 244 (27·7%)	..
>45–65	81 953	6976 (41·9%)	74 977 (42·4%)	..
>65–75	28 730	1028 (6·2%)	27 702 (15·7%)	..
>75	19 760	225 (1·4%)	19 535 (11·0%)	..
Unknown	3888	290	3598	..
**Sex**				
Male	91 419	6271 (37·2%)	85 148 (47·3%)	<0·0001
Female	105 476	10 603 (62·9%)	94 873 (52·7%)	..
Unknown	584	81	503	..
**Obesity (BMI >30 kg/m^2^)**				
Yes	571	23 (18·0%)	548 (21·4%)	0·350
No	2114	105 (82·0%)	2009 (78·6%)	..
Unknown	194 794	16 827	177 967	..
**Number of underlying conditions**				
None	102 621	6019 (39·3%)	96 602 (54·0%)	<0·0001
1–2	81 043	8075 (52·7%)	72 968 (40·8%)	..
≥3	10 574	1242 (8·1%)	9332 (5·2%)	..
Unknown	3241	1619	1622	..
**Hypertension**				
Yes	64 094	4858 (33·3%)	59 236 (33·4%)	0·802
No	127 675	9718 (66·7%)	117 957 (66·6%)	..
Unknown	5710	2379	3331	..
**Diabetes**				
Yes	44 417	3165 (22·3%)	41 252 (23·2%)	<0·05
No	147 757	11 041 (77·7%)	136 716 (76·8%)	..
Unknown	5305	2749	2556	..
**Chronic neurological disorder**				
Yes	864	22 (6·3%)	842 (4·9%)	0·241
No	16 720	330 (93·8%)	16 390 (95·1%)	..
Unknown	179 895	16 603	163 292	..
**Current smoking**				
Yes	2538	433 (8·8%)	2105 (5·2%)	<0·0001
No	43 233	4501 (91·2%)	38 732 (94·9%)	..
Unknown	151 708	1202	139 687	..
**Asthma**				
Yes	9590	912 (6·9%)	8678 (4·9%)	<0·0001
No	180 585	12 224 (93·0%)	168 361 (95·1%)	..
Unknown	7304	3819	3485	..
**Chronic kidney disease**				
Yes	4721	683 (5·4%)	4038 (2·3%)	<0·0001
No	181 438	11 920 (94·6%)	169 518 (97·7%)	..
Unknown	11 320	4352	6968	..
**Chronic pulmonary disease**				
Yes	6983	797 (6·3%)	6186 (3·6%)	<0·0001
No	178 967	11 807 (93·7%)	167 160 (96·4%)	..
Unknown	11 529	4351	7178	..
**Chronic cardiac disease**				
Yes	6206	532 (4·2%)	5674 (3·3%)	<0·0001
No	180 715	12 201 (95·9%)	168 514 (96·8%)	..
Unknown	10 558	4222	6336	..
**Chronic liver disease**				
Yes	190	83 (11·0%)	107 (0·8%)	<0·0001
No	14 270	670 (89·1%)	13 600 (99·2%)	..
Unknown	183 019	16 202	166 817	..
**Tuberculosis**				
Yes	6672	3168 (24·2%)	3504 (2·0%)	<0·0001
No	176 518	9911 (75·8%)	166 607 (98·0%)	..
Unknown	14 289	3876	10 413	..
**Malignant neoplasm**				
Yes	1380	196 (1·6%)	1184 (0·7%)	<0·0001
No	179 680	11 938 (98·4%)	167 742 (99·3%)	..
Unknown	16 419	4821	11598	..
**Severity of illness on admission**				
Mild or moderate illness	124 761	10 182 (61·6%)	114 579 (64·7%)	<0·0001
Severe or critical illness	69 118	6339 (38·4%)	62 779 (35·4%)	..
Unclassified	3602	434	3168	..
**ICU admission during hospital stay**				
Yes	4226	366 (3·2%)	3860 (6·8%)	<0·0001
No	64 051	11 139 (96·8%)	52 912 (93·2%)	..
Unknown	129 204	5450	123 754	..
**Outcome**				
Death	40 673	3913 (24·3%)	36 760 (21·7%)	<0·0001
Survived	144 510	12 166 (75·7%)	132 344 (78·3%)	..
Transferred	3364	567	2797	..
**Use of corticosteroids**				
Yes	9773	1229 (7·5%)	8544 (4·8%)	<0·0001
No	184 448	15 232 (92·5%)	169 216 (95·2%)	..
Unknown	3260	494	2766	..
**Use of systemic anticoagulants**				
Yes	3761	121 (40·1%)	3640 (31·0%)	<0·0001
No	8280	181 (59·9%)	8099 (69·0%)	..
Unknown	185 440	16 653	168 787	..

Data are n (%) or n, unless otherwise stated. BMI=body-mass index. ICU=intensive care unit. Column percentages are calculated excluding unknown or unspecified categories.

Among people living with HIV, the three most frequent symptoms were cough (62·1%), fever (55·7%), and shortness of breath (51·9%). All clinical signs and symptoms, except fever and sore throat, were more frequent among people living with HIV compared with people who were HIV-negative (appendix 2 p 3). Creatinine and bilirubin were more frequently elevated in people living with HIV, compared with individuals who were HIV-negative (creatinine 51·7% *vs* 44·4%; bilirubin 55·9% *vs* 28·5%) while the frequency of abnormal levels of D-dimer, ferritin, erythrocyte sedimentation rate, C-reactive protein, and alanine aminotransferase or serum glutamic pyruvic transaminase were similar among the two groups (appendix 2 p 4). In contrast, compared with people who were living with HIV, IL-6 was higher among individuals who were HIV-negative (42·5% *vs* 66·4%; p<0·0001), as was lactate (68·5% *vs* 90·9%; p<0·0001).

The use of corticosteroids and anticoagulants was more frequent among people living with HIV compared with HIV-negative individuals (corticosteroids 7·5% *vs* 4·8%; anticoagulants 40·1% *vs* 31·0%). In the subgroup of patients with severe presentation, corticosteroids administration remained more frequent in people living with HIV (13·2%), compared with individuals who were HIV-negative (8·1%).

38·4% of people living with HIV were admitted to the hospital in severe or critical condition and 24·3% died (table 1). People living with HIV with severe or critical disease were more likely to be older than 45 years and male (p<0·05; appendix 2 p 5), than individuals with mild or moderate presentation at admission. People with severe COVID-19 were also more likely to have diabetes, hypertension, malignancies, cardiac disease, and kidney disease (p<0·0001). The frequency of ICU admission and case-fatality rates were significantly higher in the severe or critical group, compared with the milder group (ICU admission 5·5% *vs* 0·8%; case-fatality rate 37·3% *vs* 17·1%; p<0·0001 for both).

Compared with people living with HIV who were discharged alive, individuals who died in hospital were more likely to be older than 45 years and male. Individuals who died in hospital, compared with individuals who were discharged, had significantly higher proportions of diabetes (32·9% *vs* 19·4%), hypertension (43·8% *vs* 30·1%), tuberculosis (28·3% *vs* 22·9%), chronic kidney disease (10·1% *vs* 4·1%), and malignancies (2·7% *vs* 1·3%; all p<0·0001). Having asthma, chronic cardiac, pulmonary and neurological conditions, and current smoking were not significantly different between groups. People living with HIV who died were more likely to be admitted with severe or critical disease than those with mild or moderate disease (p<0·0001; table 2).

**Table 2:** Characteristics, underlying conditions, therapeutics, and outcomes among people living with HIV hospitalised with suspected or confirmed COVID-19, by outcome status

	**Total**	**Death (n=3913)**	**Discharged alive (n=12 166)**	**p value**
**Age group, years**				
≤18	232	34 (0·8%)	198 (1·7%)	<0·0001
>18–45	7736	1328 (34·2%)	6408 (53·8%)	..
>45–65	6632	1977 (50·9%)	4655 (39·1%)	..
>65–75	977	446 (11·5%)	531 (4·5%)	..
>75	214	100 (2·6%)	114 (0·9%)	..
Unknown	288	28	260	..
**Sex**				
Male	5922	1684 (43·1%)	4238 (35·1%)	<0·0001
Female	10 087	2223 (56·9%)	7864 (64·9%)	..
Unknown	70	6	64	..
**Obesity (BMI >30 kg/m^2^)**				
Yes	20	5 (29·4%)	15 (14·4%)	0·122
No	101	12 (70·6%)	89 (85·6%)	..
Unknown	15 958	3896	12 062	..
**Corticosteroid use during hospitalisation**				
Yes	1141	375 (9·7%)	766 (6·5%)	<0·0001
No	14 485	3470 (90·3%)	11 015 (93·5%)	..
Unknown	453	68	385	..
**Systemic anticoagulant use during hospitalisation**				
Yes	94	26 (59·1%)	68 (32·5%)	<0·0001
No	159	18 (40·9%)	141 (67·5%)	..
Unknown	15 826	3869	11 957	..
**Chronic cardiac disease**				
Yes	501	127 (4·6%)	374 (3·9%)	0·113
No	11 670	2607 (95·4%)	9063 (96·1%)	..
Unknown	3908	1179	2729	..
**Diabetes**				
Yes	3042	1026 (32·9%)	2016 (19·4%)	<0·0001
No	10 470	2093 (67·1%)	8377 (80·6%)	..
Unknown	2567	794	1773	..
**Hypertension**				
Yes	4606	1422 (43·8%)	3184 (30·1%)	<0·0001
No	9250	1825 (56·2%)	7425 (69·9%)	..
Unknown	2223	666	1557	..
**Current smoking**				
Yes	397	108 (9·5%)	289 (8·4%)	0·251
No	4166	1025 (90·5%)	3141 (91·6%)	..
Unknown	11 516	2780	8736	..
**Chronic pulmonary disease**				
Yes	781	177 (6·5%)	604 (6·5%)	0·871
No	11 274	2527 (93·5%)	8747 (93·5%)	..
Unknown	4024	1209	2815	..
**Tuberculosis**				
Yes	3001	794 (28·3%)	2207 (22·9%)	<0·0001
No	9432	2017 (71·7%)	7415 (77·1%)	..
Unknown	3646	1102	2544	..
**Asthma**				
Yes	890	181 (6·5%)	709 (7·3%)	0·186
No	11 640	2589 (93·5%)	9051 (92·7%)	..
Unknown	3549	1143	2406	..
**Chronic kidney disease**				
Yes	658	275 (10·1%)	383 (4·1%)	<0·0001
No	11 393	2449 (89·9%)	8944 (95·9%)	..
Unknown	4028	1189	2839	..
**Malignant neoplasm**				
Yes	183	70 (2·7%)	113 (1·3%)	<0·0001
No	11 411	2569 (97·3%)	8842 (98·7%)	..
Unknown	4485	1274	3211	..
**Chronic liver disease**				
Yes	81	11 (11·0%)	70 (11·6%)	0·851
No	620	89 (89·0%)	531 (88·3%)	..
Unknown	15 378	3813	11 565	..
**Chronic neurological disorder**				
Yes	19	5 (9·4%)	14 (5·5%)	0·281
No	288	48 (90·6%)	240 (94·5%)	..
Unknown	15 772	3860	11 912	..
**Comorbidity burden**				
None	5725	946 (27·1%)	4779 (43·2%)	<0·0001
1–2	7623	2171 (62·2%)	5452 (49·3%)	..
≥3	1208	371 (10·7%)	837 (7·5%)	..
Unknown	1523	425	1098	..
**Severity of illness on admission**				
Mild or moderate illness	9778	1675 (43·3%)	8103 (68·7%)	<0·0001
Severe or critical illness	5889	2196 (56·7%)	3693 (31·3%)	..
Unknown	16 413	3931	12 482	..
**Admission to ICU**				
Yes	344	227 (7·1%)	117 (1·5%)	<0·0001
No	10 600	2992 (92·9%)	7608 (98·5%)	..
Unknown	5135	694	4441	..

ART=antiretroviral therapy. BMI=body-mass index. ICU=intensive care unit. Column percentages are calculated excluding unknown categories.

Risk factors for in-hospital mortality and severity were determined in the hospitalised population. After adjusting for age, sex, disease severity at admission, and underlying conditions (ie, diabetes, tuberculosis, chronic kidney diseases, pulmonary diseases, and malignancies), patients with HIV were 38% more likely to die than individuals without HIV (aHR 1·38, 95% CI 1·34–1·41). Other significant risk factors independently associated with mortality were male sex, severe or critical presentation, tuberculosis, diabetes, malignant neoplasms, chronic pulmonary disease, and kidney disease (table 3). Increasing age over 18 years showed a gradually and consistently elevated mortality risk.

**Table 3:** Risk factors for in-hospital mortality in the overall sample of patients hospitalised with suspected or confirmed COVID-19

	**Hazard ratio (95% CI)**	**p value**
**HIV status**		
HIV-negative (ref)	..	..
HIV positive	1·38 (1·34–1·41)	<0·0001
**Sex**		
Female (ref)	..	..
Male	1·07 (1·05–1·09)	<0·0001
**Age group, years**		
≤18 years (ref)	..	..
>18–45	2·50 (2·28–2·74)	<0·0001
>45–65	5·04 (4·32–5·89)	<0·0001
>65–75	7·94 (6·72–9·37)	<0·0001
>75 years	10·06 (8·36–12·10)	<0·0001
**Severity at admission**		
Mild or moderate (ref)	..	..
Severe or critical	1·37 (1·32–1·42)	<0·0001
**Tuberculosis**		
None (ref)	..	..
Tuberculosis	1·10 (1·04–1·16)	0·003
**Diabetes**		
None (ref)	..	..
Diabetes	1·39 (1·36–1·43)	<0·0001
**Chronic pulmonary disease**		
None (ref)	..	..
Chronic pulmonary disease	1·03 (1·01–1·05)	0·02
**Chronic kidney disease**		
None (ref)	..	..
Chronic kidney disease	1·58 (1·56–1·61)	<0·0001
**Malignant neoplasms**		
None (ref)	..	..
Malignant neoplasms	1·14 (1·09–1·20)	<0·0001

Covariates that did not pass covariate selection criteria and not included in the full model: asplenia, asthma, chronic cardiac, liver and neurological diseases, smoking, hypertension, obesity, antiretroviral therapy, and steroid use.

HIV infection was associated with 15% increased odds of severe or critical presentation (aOR 1·15, 95% CI 1·10–1·20) compared with individuals who were HIV-negative, after adjusting for age, sex, diabetes, tuberculosis, malignant neoplasms, chronic kidney disease, cardiac disease, pulmonary disease, and corticosteroid use. Other factors associated with severe or critical presentation were age older than 45 years (compared with individuals <18 years), chronic cardiac disease, diabetes, and malignancy (appendix 2 p 7). The increased odds of severe or critical disease and risk of mortality in people living with HIV compared with non-HIV were consistent in models adjusting for underlying condition burden (number of underlying conditions, rather than individual conditions; appendix 2 pp 8–9).

Among people with severe presentation, the median time from hospital admission to death was shorter in people living with HIV (19 days, IQR 6–49) compared with HIV-negative individuals (23 days, 10–51; p<0·0001). Conversely, among people with mild or moderate presentation, the median time from hospital admission to mortality was longer in people living with HIV (80 days, 14–not estimable) compared with people who were HIV-negative (32 days, 15–102; p<0·0026).

Overall, 9773 (5%) of 194 221 patients had received corticosteroids during hospitalisation. Of those, 1229 (7·5%) were people living with HIV and 8544 (4·8%) were HIV-negative (p<0·0001). Among patients with severe COVID-19 who received corticosteroids, people living with HIV were more likely to die compared with people who were HIV-negative (aHR 1·25, 95% CI 1·04–1·50).

Three exploratory subgroup analyses were done to assess the impact of geographical region, ART use, and viral load status on mortality and severity. Compared with people who were HIV-negative, people living with HIV were more likely to die from COVID-19 in the WHO African region (aHR 1·28, 95% CI 1·24–1·33), but not in the WHO European region (aHR 1·50, 0·77–2·94) or WHO region of the Americas (aHR 1·18, 0·76–1·82), after adjusting for age, sex, underlying conditions, and clinical presentation.

In an exploratory subgroup analysis assessing the effect of ART on mortality in a subset of 9097 patients from South Africa reporting ART information, both people living with HIV on ART (aHR 1·48, 95% CI 1·39–1·57) and not on ART (aHR 1·79, 1·48–2·16) had a significantly higher risk of death compared with people who were HIV-negative.

A similar exploratory analyses on a sample of 5793 patients from South Africa reporting viral load information (68·5% viral load of <1000 copies per mL, 31·5% viral load of >1000 copies per mL) showed that the risk of death was equally greater among people living with HIV with viral load of more than 1000 copies per mL (aHR 1·77, 1·57–1·99) and in those with viral load of less than 1000 copies per mL (aHR 1·45, 1·32–1·58) compared with individuals who were HIV-negative.

Finally, risk factors for in-hospital mortality and severity among people living with HIV were then determined. Among people living with HIV the most significant risk factor for in-hospital mortality was severe or critical presentation (aHR 1·86, 95% CI 1·82–1·90), followed by chronic kidney disease, diabetes, malignant neoplasms, tuberculosis, male sex, and hypertension. Increase in age category was associated with increased mortality risk (table 4). Among people living with HIV, factors significantly associated with severe or critical COVID-19 at admission were chronic cardiac disease, male sex, and age 45–75 years (appendix 2 p 10).

**Table 4:** Risks factors for in-hospital mortality among people living with HIV hospitalised with suspected or confirmed COVID-19

	**Hazard ratio (95% CI)**	**p value**
**Sex**		
Female (ref)	..	..
Male	1·11 (1·04–1·18)	0·0061
**Age group, years**		
≤18 (ref)	..	..
>18–45	1·50 (1·06–2·14)	0·0287
>45–65	2·34 (1·65–3·32)	0·0002
>65–75	3·47 (2·35–5·12)	<0·0001
>75	4·09 (2·72–6·18)	<0·0001
**Severity at admission**		
Mild or moderate (ref)	..	..
Severe or critical	1·86 (1·82–1·90)	<0·0001
**Tuberculosis**		
None (ref)	..	..
Tuberculosis	1·15 (1·10–1·20)	<0·0001
**Diabetes**		
None (ref)	..	..
Diabetes	1·36 (1·30–1·43)	<0·0001
**Hypertension**		
None (ref)	..	..
Hypertension	1·06 (1·04–1·09)	<0·0001
**Chronic kidney disease**		
None (ref)	..	..
Chronic kidney disease	1·41 (1·31–1·52)	<0·0001
**Malignant neoplasms**		
None (ref)	..	..
Malignant neoplasms	1·24 (1·12–1·38)	0·0003

Covariates that did not pass covariate selection criteria and not included in the full model: asplenia, asthma, chronic cardiac, liver, neurological and pulmonary diseases, smoking, obesity, antiretroviral therapy, and steroid use.

In an exploratory analysis assessing the impact of ART and viral load on clinical outcomes, people living with HIV on ART were 17% less likely to die (p=0·048) and 40% less likely to be admitted with severe disease than those not on ART (p<0·0001). Among people living with HIV, individuals with viral load of less than 1000 copies per mL were 15% less likely to die while in the hospital and 45% less likely to be admitted with severe disease compared with those with viral load of more than 1000 copies per mL (p<0·0001).

## Discussion

This analysis found that people living with HIV had 15% greater odds of being admitted to hospital with a severe or critical COVID-19 presentation and, once hospitalised, were 38% more likely to die in hospital than people who were HIV-negative. Among people with severe presentation, the median time from hospital admisson to death was shorter in people living with HIV. Among people hospitalised with COVID-19, underlying conditions were more frequent in people living with HIV than people who were HIV-negative, putting them at increased risk of poor outcomes. We found that older age, tuberculosis, and pulmonary diseases were associated with increased risk of in-hospital mortality. Other factors associated with an increased risk of mortality were diabetes, kidney diseases, and malignancies, in line with other reports.^10^, ^26^ Our exploratory analyses showed that the use of ART or viral load suppression were associated with a reduced risk of poor outcomes among people living with HIV; however, HIV infection remained a risk factor for severity and mortality regardless of ART and viral load suppression status.

The independent association between HIV infection and poor COVID-19 outcomes suggests that the mechanisms for the more severe COVID-19 course in people living with HIV might reside with the HIV disease pathophysiology. T-helper-cell lymphopenia is known to contribute to immunosuppression and thus increases the risk of opportunistic infections.^29^ We also know that people living with HIV—despite adequate treatment—can have a higher propensity to chronic inflammation, underlying immune dysregulation, and cytokine storm due to key cytokines IL-6, IL-1, and TNF-a, and, to a lesser extent, IL-10 and GM-CSFs.^30^ The increased immune activation and persistent, chronic inflammation associated with HIV infection are major players in the accelerated aging process.

The role of immune depression or dysregulation needs to be further investigated to explain the intersection between the two infections, and the mechanisms behind the increased risk of severe or fatal COVID-19 in people living with HIV.

WHO recommends the use of corticosteroids in severely ill COVID-19 patients.^28^ In this sample, corticosteroid use was infrequent in both people living with HIV and people who were HIV-negative with severe presentation, indicating the need to increase the access to this therapeutic. We also found that tuberculosis and pulmonary diseases were associated with increased risk of in-hospital mortality, but not of severe presentation; this inconsistency might be explained by selection bias in the criteria or timing of hospitalisation (ie, people with tuberculosis or pulmonary diseases with initial symptoms of COVID-19 might access the hospital earlier or be triaged for an early admission, thus reducing the likelihood of severe presentation at admission). Of note, in our dataset, severity status was assessed within the first 24 h of hospital admission.

Our findings have important public health implications. We found that underlying conditions are common and more frequent among people living with HIV than the general population. Alongside the response to COVID-19, it is thus critical to maintain access to essential health services for this vulnerable group. These include supporting people living with HIV to stay as healthy as possible, regularly access and take their ART medications to achieve viral load suppression, and prevent, diagnose, and manage underlying conditions and co-infections. HIV infection cannot be adequately managed if it is not diagnosed in the first place. Concerningly, large decreases in HIV testing services have been seen across countries. The Global Fund reported that HIV testing declined by 41% in LMICs and referrals for diagnosis and treatment declined by 37% between April and September, 2020, compared with the same period in 2019.^31^ Concerted efforts are needed to put in place an HIV testing catch-up plan. The increased risk of poor outcomes in people living with HIV hospitalised for COVID-19 should prompt countries to consider including this population as a priority group for COVID-19 vaccination according to the epidemiological context. Informed by these findings, the WHO Strategic Advisory Group of Experts on Immunization (SAGE) has included people living with HIV as a risk group requiring priority consideration for vaccination of COVID-19 in the context of limited supply.^32^ Countries are already moving in this direction: an informal WHO survey of 100 countries found that 40 have an immunisation policy that prioritises vaccination for people living with HIV.^33^

Our analysis has several strengths. We were able to pool patient-level data from a large number of facilities using standardised data collection tools, providing sufficient statistical power to support a number of different analyses to assess risk factors for adverse outcomes. There are also several limitations to note. Not all facilities completed all the variables included in the WHO CRF, resulting in some data missingness, including laboratory markers, which were found to influence outcomes in other studies.^34^ Only documented covariates could be assessed as risk factors; in particular, data on CD4 cell counts were unavailable for this analysis. Lack of information on CD4 cell count or previous AIDS events was an important limitation, as it precluded ascertaining the role of immunodepression in the evolution of COVID-19. Differences in key socioeconomic determinants of health related to HIV status, such as income and food insecurity, could partly explain adverse outcomes, but this could not be assessed because appropriate indicators were not collected. Similarly, information on ART and viral load use was available in a subgroup of patients, and information on other COVID-19 therapeutics was even more sparse, thus precluding meaningful analysis of clinical outcomes stratified by medication use. WHO is currently updating the CRF to gather this information to inform future analyses. Some countries contributed data derived from national registries, while others from a convenience sample of hospitals or sentinel clinics, thus with potential reporting biases. In addition, most data were from Africa (in particular South Africa), potentially limiting the generalisability of these findings to other contexts. In particular, hospitalisation criteria, concomitant co-infections, COVID-19 severity at admission, immunodeficiency, use of ART and access to health care can be expected to vary by country. Nevertheless, it is worth noting that 65% of the HIV cases globally are from Africa, and South Africa hosts the largest HIV epidemic in the world, suggesting the applicability of the findings as a minimum in the region with the highest HIV burden. Lastly, although information on vaccination status were unavailable in this sample, the vaccination coverage in LMICs was very low at the time of these data collection (approximately 1–2%, as of July, 2021), and thus unlikely to play a significant role in influencing these results. Notably, as our study population includes hospitalised individuals, we could not explore factors influencing the risk of hospitalisation, including the correlation between HIV infection and COVID-19 hospitalisation.

In conclusion, we report, to our knowledge, the largest multicountry analysis to date exploring the association between HIV infection and clinical outcomes in people hospitalised with COVID-19 using individual-level data. Our results show HIV infection to be independently associated with increased odds of presenting with severe or critical disease at hospital admission and increased likelihood of in-hospital mortality. These findings have informed the WHO clinical management guidelines of COVID-19 and the WHO SAGE vaccination road map. Because our findings of an increased risk in mortality mostly reflect the large data contribution from the African region, we plan to regularly update the analysis by including datasets from other countries as they become available, and this is expected to improve the generalisability of the results. WHO continues to expand the collection of anonymised clinical data, including on other variables of interest (eg, vaccination status, re-infection, variants, therapeutics, CD4 cell counts, and viral load). This analysis will be updated regularly, and as of April 1, 2022, the WHO Platform included 621 441 total patients, of whom 37 453 were people living with HIV. WHO encourages countries and stakeholders to support the generation of evidence-based interventions, including optimised vaccination strategies for subpopulations, by contributing their data through the WHO Global Platform for COVID-19.

## Data sharing

All relevant documents related to the WHO Clinical Platform, including the statistical analysis plan, CRF, data dictionary, and terms of use, can be found on the WHO Global Clinical Platform webpage.^27^ Data submitted to the WHO Platform are the property of the individual Ministries of Health. All data outputs will be published in an open access format on the webpage.

## Declaration of interests

RH received funding from the Wellcome Trust, Canadian Institute of Health Research, UK Research and Innovation/Medical Research Council, and International COVID-19 Data Alliance–Health Data Research UK. All other authors declare no competing interests.

## Supplementary Materials

French translation of the abstract
