# Supplementary material for: Clinical features of, and risk factors for, severe or fatal COVID-19 among people living with HIV admitted to hospital: analysis of data from the WHO Global Clinical Platform of COVID-19
Source: Lancet HIV. 2022 May 10;9(7):e486–95. doi: 10.1016/S2352-3018(22)00097-2 (PMC9090268; doi:10.1016/S2352-3018(22)00097-2)
Supplement: French translation of the abstract [file mmc1.pdf]

THE LANCET
HIV

Supplementary appendix 1

This translation in French was submitted by the authors and we reproduce it as supplied. It has not been peer reviewed. *The Lancet*'s editorial processes have only been applied to the original in English, which should serve as reference for this manuscript.

Cette traduction en français a été proposée par les auteurs et nous l'avons reproduite telle quelle. Elle n'a pas été examinée par des pairs. Les processus éditoriaux du *Lancet* n'ont été appliqués qu'à l'original en anglais et c'est cette version qui doit servir de référence pour ce manuscrit.

Supplement to: Bertagnolio S, Thwin SS, Silva R, et al. Clinical features of, and risk factors for, severe or fatal COVID-19 among people living with HIV admitted to hospital: analysis of data from the WHO Global Clinical Platform of COVID-19. *Lancet HIV* 2022; published online May 10. https://doi.org/10.1016/S2352-3018(22)00097-2.

APPENDIX 1

Caractéristiques cliniques et facteurs de risque de développement d'une forme grave ou mortelle de la COVID-19 chez les personnes vivant avec le VIH admises à l'hôpital: analyse des données de la Plateforme Clinique Mondiale de l'OMS sur le COVID-19.

Contexte

L'Organisation Mondiale de la Santé (OMS) a mis en place une plateforme mondiale pour la surveillance clinique de la COVID-19 chez les personnes hospitalisées. Nous avons évalué si les personnes vivant avec le VIH et hospitalisées en raison de la COVID-19 présentaient un risque accru de développement d'une forme grave de la COVID-19 et de mortalité par rapport aux personnes séronégatives pour le VIH et les facteurs de risque associés.

Entre le 1er janvier 2020 et 1 juillet 2021, les données individuelles et anonymes de 338 566 patients provenant de 38 pays ont été partagées avec l'OMS. En s'appuyant sur l'ensemble des données collectées, nous avons effectué des statistiques descriptives et des analyses de régression afin de comparer les pronostics cliniques dans les deux populations et d'identifier les facteurs de risque.

Sur 197 479 patients connaissant leur statut VIH, 16 955 (8,6 %) vivaient avec le VIH; 16283 (96 %) venaient d'Afrique; 10603 (62.9 %) étaient des femmes et 6271 (37,1 %) étaient des hommes; l'âge moyen était de 45,5 ans (SD 13.7); 6339 (38,3 %) ont été admis à l'hôpital avec une forme clinique grave et 3913 (24,3 %) sont décédés à l'hôpital. Parmi les 10166 personnes vivant avec le VIH et dont l'information concernant la prise du traitement antirétroviral (TAR) était connue, 9302 (91,5%) étaient sous TAR. En comparaison avec les personnes séronégatives pour le VIH, les personnes vivant avec le VIH présentaient un risque d'être admis à l'hôpital avec des symptômes graves de la COVID-19 (RCA 1,15, IC à 95 % 1,10-1,20) plus élevé de 15 % et un risque de mortalité à l'hôpital (RH 1,38, 1,34-1,41) plus élevé de 38 %. Chez les personnes vivant avec le VIH de sexe masculin, âgé de 45 à 75 ans, et ayant une maladie cardiaque chronique ou d'hypertension, le risque de développer une forme grave de la COVID-19 augmentait; le sexe masculin, le fait d'être âgé de plus de 18 ans, le diabète, l'hypertension, les cancers, la tuberculose ou les maladies rénales chroniques augmentaient le risque de mortalité à l'hôpital. L'utilisation du TAR ou la suppression de la charge virale conduisait à réduire le risque de mortalité; toutefois, l'infection par le VIH demeurait un facteur de risque de développer une forme grave de la COVID-19 ou d'en décéder, quel que soit l'utilisation de la TAR et la suppression de la charge virale.

Interprétation

Dans cet échantillon de personnes hospitalisées dont les données ont été transmises à la Plateforme Clinique Mondiale de l'OMS pour la COVID-19, le VIH était un facteur de risque indépendant à la fois de développement d'une forme grave de la COVID-19 et de décès à l'hôpital. Ces résultats ont éclairé les stratégies de vaccination de l'OMS qui donne la priorité à la vaccination des personnes vivant avec le VIH. Comme les résultats reflètent principalement des données d'Afrique, cette analyse sera mise à jour au fur et à mesure que des données d'autres régions seront disponibles.
