# Supplementary material for: Clinical features of, and risk factors for, severe or fatal COVID-19 among people living with HIV admitted to hospital: analysis of data from the WHO Global Clinical Platform of COVID-19
Source: Lancet HIV. 2022 May 10;9(7):e486–95. doi: 10.1016/S2352-3018(22)00097-2 (PMC9090268; doi:10.1016/S2352-3018(22)00097-2)
Supplement: Supplementary appendix 2 [file mmc2.pdf]

THE LANCET
HIV

Supplementary appendix 2

This appendix formed part of the original submission and has been peer reviewed. We post it as supplied by the authors.

Supplement to: Bertagnolio S, Thwin SS, Silva R, et al. Clinical features of, and risk factors for, severe or fatal COVID-19 among people living with HIV admitted to hospital: analysis of data from the WHO Global Clinical Platform of COVID-19. *Lancet HIV* 2022; published online May 10. https://doi.org/10.1016/S2352-3018(22)00097-2.

Appendix of Supplementary Tables

Table of Contents

- Supplementary Table 1:
  List of countries contributing clinical data of >30 patients hospitalized with COVID-19.
- Supplementary Table 2:
  Presenting symptoms among people hospitalized with suspected or confirmed COVID-19, by HIV status
- Supplementary Table 3:
  Laboratory markers among PLHIV and HIV negative individuals hospitalized with suspected or confirmed COVID-19
- Supplementary Table 4:
  Characteristics, underlying conditions, therapeutics, and outcomes of people living with HIV hospitalized with suspected or confirmed COVID-19, by severity of illness at time of admission
- Supplementary Table 5:
  Factors associated with severe/critical illness in the overall sample of patients hospitalized with suspected or confirmed COVID-19
- Supplementary Table 6:
  Factors associated with severe/critical illness in hospitalized patients with suspected or confirmed COVID-19
- Supplementary Table 7:
  Risk factors of in-hospital mortality in hospitalized patients with suspected or confirmed COVID-19
- Supplementary Table 8:
  Factors associated with severe/critical presentation among people living with HIV hospitalized with suspected or confirmed COVID-19

Supplementary Table 1: List of countries contributing clinical data of >30 patients hospitalized with COVID-19.

	Total	HIV	
		Yes (n=16955)	No (n=180524)
**Country**			
Brazil	5382	69 (0.41%)	5313 (2.94%)
Burkina Faso	197	NA	197 (0.11%)
Cameroon	1229	31 (0.18%)	1198 (0.66%)
Chile	56	11 (0.06%)	45 (0.02%)
Colombia	252	NA	252 (0.14%)
Democratic Republic of Congo	210	2 (0.01%)	208 (0.12%)
Dominican Republic	257	7 (0.04%)	250 (0.14%)
Germany	105	1 (0.01%)	104 (0.06%)
Ghana	712	NA	712 (0.39%)
Guinea	1023	16 (0.09%)	1007 (0.56%)
India	397	8 (0.05%)	389 (0.22%)
Italy	2111	117 (0.69%)	1994 (1.10%)
Jordan	498	1 (0.01%)	497 (0.28%)
Niger	112	2 (0.01%)	110 (0.06%)
Nigeria	2607	23 (0.14%)	2584 (1.43%)
Panama	259	4 (0.2%)	255 (0.14%)
Republic of Korea	99	NA	99 (0.05%)
Russia	65	10 (0.06%)	55 (0.03%)
Saudi Arabia	133	NA	133 (0.07%)
Singapore	124	NA	124 (0.07%)
South Africa	175748	16008 (94.41%)	159740 (88.49%)
Spain	4038	89 (0.52%)	3949 (2.19%)
USA	664	300 (1.77%)	364 (0.20%)
United Kingdom	37	36 (0.21%)	1 (0.00%)
Zambia	80	23 (0.14%)	57 (0.03%)
Zimbabwe	993	178 (1.05%)	815 (0.45%)

Data in table represent contributions stemming from countries that have submitted greater than 30 overall cases.
NA = not available (no data reported to the platform).

Supplementary Table 2. Presenting symptoms among people hospitalized with suspected or confirmed COVID-19, by HIV status

	Total	PLHIV (n=16955)	HIV negative (n= 180524)	p-value
**History of fever**				
Yes	9176	290 (55.7%)	8886 (52.3%)	0.14
No	8321	231 (44.3%)	8090 (47.7%)	
Unknown	*4234*	*426*	*3808*	
**Cough**				
Yes	7818	272 (62.1%)	7546 (50.4%)	<0.0001
No	7594	166 (37.9%)	7428 (49.6%)	
Unknown	*6319*	*509*	*5810*	
**Shortness of breath**				
Yes	3703	180 (51.9%)	3523 (32.8%)	<0.0001
No	7386	167 (48.1%)	7219 (67.2%)	
Unknown	*10642*	*600*	*10042*	
**Fatigue / Malaise**				
Yes	3428	174 (45.4%)	3254 (30.2%)	<0.0001
No	7718	209 (54.6%)	7509 (69.8%)	
Unknown	*10585*	*564*	*10021*	
**Headache**				
Yes	2657	96 (22.0%)	2561 (14.1%)	<0.0001
No	15901	341 (78.0%)	15560 (85.9%)	
Unknown	*3173*	*510*	*2663*	
**Sore throat**				
Yes	1438	37 (10.0%)	1401 (12.6%)	0.14
No	10077	333 (90.0%)	9744 (87.4%)	
Unknown	*10216*	*577*	*9639*	
**Myalgia**				
Yes	2158	85 (18.2%)	2073 (11.4%)	<0.0001
No	16460	382 (81.8%)	16078 (88.6%)	
Unknown	*3113*	*480*	*2633*	
**Chest pain**				
Yes	1343	77 (19.4%)	1266 (9.4%)	<0.0001
No	12460	319 (80.6%)	12141 (90.6%)	
Unknown	*7928*	*551*	*7377*	
**Diarrhoea**				
Yes	1379	78 (16.1%)	1301 (8.5%)	<0.0001
No	14369	407 (83.9%)	13962 (91.5%)	
Unknown	*5983*	*462*	*5521*	
**Loss of smell**				
Yes	1064	32 (11.9%)	1032 (8.3%)	<0.05
No	11707	238 (88.1%)	11469 (91.7%)	
Unknown	*8960*	*677*	*8283*	

PLHIV= people living with HIV; Column percentages are calculated excluding Unknown/Unspecified categories.

Supplementary Table 3. Laboratory markers among PLHIV and HIV negative individuals hospitalized with suspected or confirmed COVID-19

	Total	PLHIV (n=16955)	HIV negative (n= 180524)	p-value
**D-Dimer > 1 mg/L**				
Yes	3409	119 (77.8%)	3290 (79.9%)	0.52
No	861	34 (22.2%)	827 (20.1%)	
Unknown	*193211*	*16802*	*176409*	
**Ferritin > 300 ng/ml**				
Yes	1674	79 (92.9%)	1595 (90.0%)	0.37
No	184	6 (7.1%)	178 (10.0%)	
Unknown	*195623*	*16870*	*178753*	
**ESR > 100 mm/hour**				
Yes	36	1 (2.50%)	35 (5.7%)	0.39
No	614	39 (97.6%)	575 (94.3%)	
Unknown	*196831*	*16915*	*179916*	
**CRP > 100 mg/l**				
Yes	6298	124 (79.0%)	6174 (81.6%)	0.39
No	1423	33 (21.0%)	1390 (18.4%)	
Unknown	*189760*	*16798*	*172962*	
**IL-6 > 10 pg/ml**				
Yes	551	23 (42.5%)	528 (66.4%)	<0.001
No	298	31 (57.5%)	267 (33.6%)	
Unknown	*196632*	*16901*	*179731*	
**Bilirubin > 2 mg/dl**				
Yes	1524	52 (55.9%)	1472 (28.5%)	<0.0001
No	3740	41 (44.1%)	3699 (71.5%)	
Unknown	*192217*	*16862*	*175355*	
**ALT/SGPT > 50 U/L**				
Yes	1750	30 (18.2%)	1720 (24.8%)	0.05
No	5363	135 (81.8%)	5228 (75.2%)	
Unknown	*190368*	*16790*	*173578*	
**Creatinine > 1.2 mg/dL**				
Yes	4118	107 (51.7%)	4011 (44.4%)	<0.05
No	5114	100 (48.3%)	5014 (55.6%)	
Unknown	*188249*	*16748*	*171501*	
**Lactate > 2.2 mmol/L**				
Yes	1911	50 (68.5%)	1861 (90.9%)	<0.0001
No	210	23 (31.5%)	187 (9.1%)	
Unknown	*195360*	*16882*	*178478*	

PLHIV= people living with HIV; Column percentages are calculated excluding Unknown/Unspecified categories.

Supplementary Table 4. Characteristics, underlying conditions, therapeutics, and outcomes of people living with HIV hospitalized with suspected or confirmed COVID-19, by severity of illness at time of admission

	Total	Severe/Critical (n= 6339)	Mild/Moderate (n = 10182)	p-value
**Age Group**				
<=18yr	257	92 (1.5%)	165 (1.6%)	<0.0001
>18 to 45yrs	8134	2431 (38.4%)	5703 (56.1%)	
>45 to 65yrs	6896	3169 (50.0%)	3727 (36.3%)	
>65 to 75yrs	1010	534 (8.4%)	476 (4.7%)	
>75yrs	215	111 (1.8%)	104 (1.0%)	
Unknown	*9*	*2*	*7*	
**Sex**				
Male	5978	2474 (39.1%)	3504 (34.6%)	<0.0001
Female	10482	3849 (60.9%)	6633 (65.4%)	
Unknown	*61*	*16*	*45*	
**Obesity (BMI>30)**				
Yes	17	9 (17.7%)	8 (22.9%)	0.55
No	69	42 (82.3%)	27 (77.1%)	
Unknown	*16435*	*6288*	*10147*	
**Chronic cardiac disease**				
Yes	397	239 (5.7%)	158 (1.9%)	<0.0001
No	11952	3987 (94.3%)	7965 (98.1%)	
Unknown	*4172*	*2113*	*2059*	
**Diabetes**				
Yes	3041	1291 (26.2%)	1750 (19.8%)	<0.0001
No	10747	3640 (73.8%)	7107 (80.2%)	
Unknown	*2733*	*1408*	*1325*	
**Hypertension**				
Yes	4662	2167 (41.8%)	2495 (27.7%)	<0.0001
No	9511	3023 (58.2%)	6488 (72.3%)	
Unknown	*2348*	*1149*	*1199*	
**Current Smoking**				
Yes	420	214 (7.9%)	206 (9.6%)	<0.05
No	4414	2483 (92.1%)	1931 (90.4%)	
Unknown	*11687*	*3642*	*8045*	
**Chronic pulmonary disease**				
Yes	742	147 (3.5%)	595 (7.4%)	<0.0001
No	11511	4039 (96.5%)	7472 (92.6%)	
Unknown	4268	2153	2115	
**Tuberculosis**				
Yes	3150	847 (18.9%)	2303 (27.1%)	<0.0001
No	9840	3636 (81.1%)	6204 (72.9%)	
Unknown	3531	1856	1675	
**Asthma**				
Yes	848	220 (5.0%)	628 (7.5%)	<0.0001
No	11931	4161 (95.0%)	7770 (92.5%)	
Unknown	3742	1958	1784	
**Chronic kidney disease**				
Yes	628	275 (6.6%)	353 (4.4%)	<0.0001
No	11597	3900 (93.4%)	7697 (96.6%)	
Unknown	4296	2164	2132	
**Malignant neoplasm**				
Yes	185	79 (1.9%)	106 (1.3%)	<0.05
No	11890	3981 (98.1%)	7909 (98.7%)	
Unknown	4446	2279	2167	
**Chronic liver disease**				
Yes	41	14 (7.3%)	27 (15.9%)	<0.05
No	320	177 (92.7%)	143 (84.1%)	
Unknown	16160	6148	10012	
**Chronic neurological disorder**				
Yes	19	11 (6.3%)	8 (6.6%)	0.90
No	278	165 (93.8%)	113 (93.4%)	
Unknown	16224	6163	10061	
**Comorbidity Burden**				
None	5902	1979 (35.4%)	3923 (42.1%)	<0.0001
1-2	7883	3279 (58.7%)	4604 (49.3%)	
>=3	1127	332 (5.9%)	795 (8.5%)	
Unknown	*1609*	*749*	*860*	
**Corticosteroid use during hospitalization**				
Yes	1186	828 (13.2%)	358 (3.5%)	<0.0001

No	15222	5462 (86.8%)	9760 (94.5%)	
Unknown	*113*	*49*	*64*	
**Systemic anticoagulant use during hospitalization**				
Yes	102	90 (57.7%)	12 (12.1%)	<0.0001
No	153	66 (42.3%)	87 (87.9%)	
Unknown	*16266*	*6183*	*10083*	
**ICU admission**				
Yes	359	316 (5.5%)	43 (0.8%)	<0.0001
No	11133	5417 (94.5%)	5716 (99.2%)	
Unknown	*5029*	*606*	*4423*	
**Outcome***				
Death	3871	2196 (37.3%)	1675 (17.1%)	<0.0001
Survived	11796	3693 (62.7%)	8103 (82.8%)	
Transferred	566	311	255	
Unknown	*288*	*139*	*149*	

PLHIV = people living with HIV; BMI = body mass index; ICU = Intensive Care Unit;
Column percentages are calculated excluding Unknown and Transferred categories.

Supplementary Table 5. Factors associated with severe/critical illness in the overall sample of patients hospitalized with suspected or confirmed COVID-19

	Odds Ratio	95% Confidence Limits		p-value
		Lower Bound	Upper Bound	
**HIV status**				
HIV negative (ref)	..	..	..	..
HIV positive	1.15	1.10	1.20	<.0001
**Sex**				
Female (ref)	..	..	..	..
Male	1.17	1.15	1.20	<.0001
**Age group**				
≤ 18 years (ref)	..	..	..	..
>18 to 45 years	1.80	1.67	1.94	<.0001
>45 to 65 years	3.19	2.96	3.43	<.0001
>65 to 75 years	3.50	3.24	3.78	<.0001
>75 years	3.50	3.23	3.78	<.0001
**Diabetes**				
None (ref)	..	..	..	..
Diabetes	1.26	1.23	1.29	<.0001
**Tuberculosis**				
None (ref)	..	..	..	..
Tuberculosis	0.52	0.48	0.55	<.0001
**Malignant Neoplasms**				
None (ref)	..	..	..	..
Malignant Neoplasms	1.66	1.47	1.88	<.0001
**Chronic Pulmonary Disease**				
None (ref)	..	..	..	..
Chronic Pulmonary Disease	0.46	0.43	0.49	<.0001
**Chronic Cardiac Disease**				
None (ref)	..	..	..	..
Chronic Cardiac Disease	2.61	2.44	2.79	<.0001
**Chronic Kidney Disease**				
None (ref)	..	..	..	..
Chronic Kidney Disease	0.66	0.61	0.70	<.0001

Covariates that did not pass covariate selection criteria and were not included in the full model**:**
asplenia, asthma, hypertension, chronic liver and neurological diseases, smoking, obesity and ART use

Supplementary Table 6: Factors associated with severe/critical illness in hospitalized patients with suspected or confirmed COVID-19

	Regression Estimates							
	**Overall**				**PLHIV**			
	**Odds Ratio**	**95% Confidence Limits**		**p-value**	**Odds Ratio**	**95% Confidence Limits**		**p-value**
		**Lower Bound**	**Upper Bound**			**Lower Bound**	**Upper Bound**	
**HIV**								
HIV negative (ref)	*1.00*	..	..	..	NA	NA	NA	NA
HIV positive	1.10	1.06	1.14	<.0001	NA	NA	NA	NA
**Sex**								
Female (ref)	*1.00*	..	..	..	*1.00*	..	..	..
Male	1.18	1.16	1.21	<.0001	1.08	1.01	1.16	0.03
**Age group**								
≤ 18 years (ref)	*1.00*	..	..	..	*1.00*	..	..	..
>18 to 45 years	1.74	1.62	1.87	<.0001	0.82	0.61	1.11	0.20
>45 to 65 years	2.98	2.78	3.19	<.0001	1.67	1.24	2.27	0.14
>65 to 75 years	3.38	3.14	3.63	<.0001	2.19	1.58	3.04	<.001
>75 years	3.63	3.37	3.92	<.0001	2.06	1.36	2.12	<.001
**Comorbidities**								
None (ref)	*1.00*	..	..	..	*1.00*	..	..	..
1-2 Comorbidities	1.65	1.61	1.68	<.0001	1.16	1.07	1.25	<.001
>=3 Comorbidities	1.18	1.13	1.23		0.61	0.53	0.70	<.001
**Administration of corticosteroids**								
None (ref)	*1.00*	..	..	..	*1.00*	..	..	..
Yes	4.89	4.65	5.16	<.0001	4.21	3.67	4.83	<.001

PLHIV = people living with HIV; Ref = reference; NA = not applicable.

Supplementary Table 7: Risk factors of in-hospital mortality in hospitalized patients with suspected or confirmed COVID-19

Regression Estimates								
	Overall				PLHIV			
	Hazard Ratio	95% Confidence Limits		p-value	Hazard Ratio	95% Confidence Limits		p-value
		Lower Bound	Upper Bound			Lower Bound	Upper Bound	
**HIV**								
HIV negative (ref)	*1.00*	..	..	..	NA	NA	NA	NA
HIV positive	1.48	1.37	1.60	<.0001	NA	NA	NA	NA
**Sex**								
Female (ref)	*1.00*	..	..	..	*1.00*	..	..	..
Male	1.05	1.08	1.10	0.023	1.11	1.05	1.16	<.001
**Age group**								
≤ 18 years (ref)	*1.00*	..	..	..	*1.00*	..	..	..
>18 to 45 years	2.54	2.37	2.72	<.0001	1.23	0.99	1.51	0.05
>45 to 65 years	5.14	4.54	5.82		1.86	1.50	2.32	<.0001
>65 to 75 years	7.99	7.11	8.97		2.77	2.14	3.58	<.0001
>75 years	9.60	8.49	10.85		3.15	2.49	3.98	<.0001
**Severity of disease**								
Mild/moderate (ref)	*1.00*	..	..	..	*1.00*	..	..	..
Severe/critical	1.34	1.22	1.47	<.0001	2.01	1.94	2.08	<.0001
**Comorbidities**								
None (ref)	*1.00*	..	..		*1.00*	..	..	
1-2 Comorbidities	1.31	1.28	1.33	<.0001	1.34	1.30	1.38	<.0001
>=3 Comorbidities	1.44	1.23	1.68		1.53	1.48	1.58	

PLHIV = people living with HIV; Ref = reference; NA = not applicable.

Supplementary Table 8. Factors associated with severe/critical presentation among people living with HIV hospitalized with suspected or confirmed COVID-19

	Odds Ratio	95% Confidence Limits		p-value
		Lower Bound	Upper Bound	
**Sex**				
Female (ref)	..	..	..	..
Male	1.14	1.05	1.24	<0.01
**Age group**				
≤ 18 years (ref)	..	..	..	..
>18 to 45 years	0.84	0.59	1.19	0.32
>45 to 65 years	1.56	1.09	2.22	<0.001
>65 to 75 years	1.95	1.33	2.86	0.02
>75 years	1.51	0.92	2.49	0.10
**Tuberculosis**				
None (ref)	..	..	..	..
Tuberculosis	0.58	0.52	0.65	<.0001
**Chronic Pulmonary Disease**				
None (ref)	..	..	..	..
Chronic Pulmonary Disease	0.32	0.25	0.40	<.0001
**Chronic Cardiac Disease**				
None (ref)	..	..	..	..
Chronic Cardiac Disease	2.96	2.28	3.83	<.0001
**Hypertension**				
None (ref)	..	..	..	..
Hypertension	1.19	1.09	1.31	<.001

Covariates that did not pass covariate selection criteria and that were not included in the full model:
asplenia, asthma, chronic kidney, liver and neurological diseases, diabetes, malignancies, smoking and obesity
